# Supplementary material for: Unraveling the Toxicological Effects of Hydroxyacetone - A Reaction Product in Electronic Cigarette Aerosols
Source: bioRxiv. 2025 Sep 4:2025.08.29.673133. Preprint. [Version 1] doi: 10.1101/2025.08.29.673133 (PMC12424699; doi:10.1101/2025.08.29.673133)

A

	Well 1 (ug/mL)	Well 2 (ug/mL)	Well 3 (ug/mL)	Well 4 (ug/mL)	Well 5 (ug/mL)	Mean ± SD (ug/mL)
**Hydroxyacetone**	**106.7**	**120.3**	**116.1**	**102.8**	**111.6**	**111.5±6.3**

B

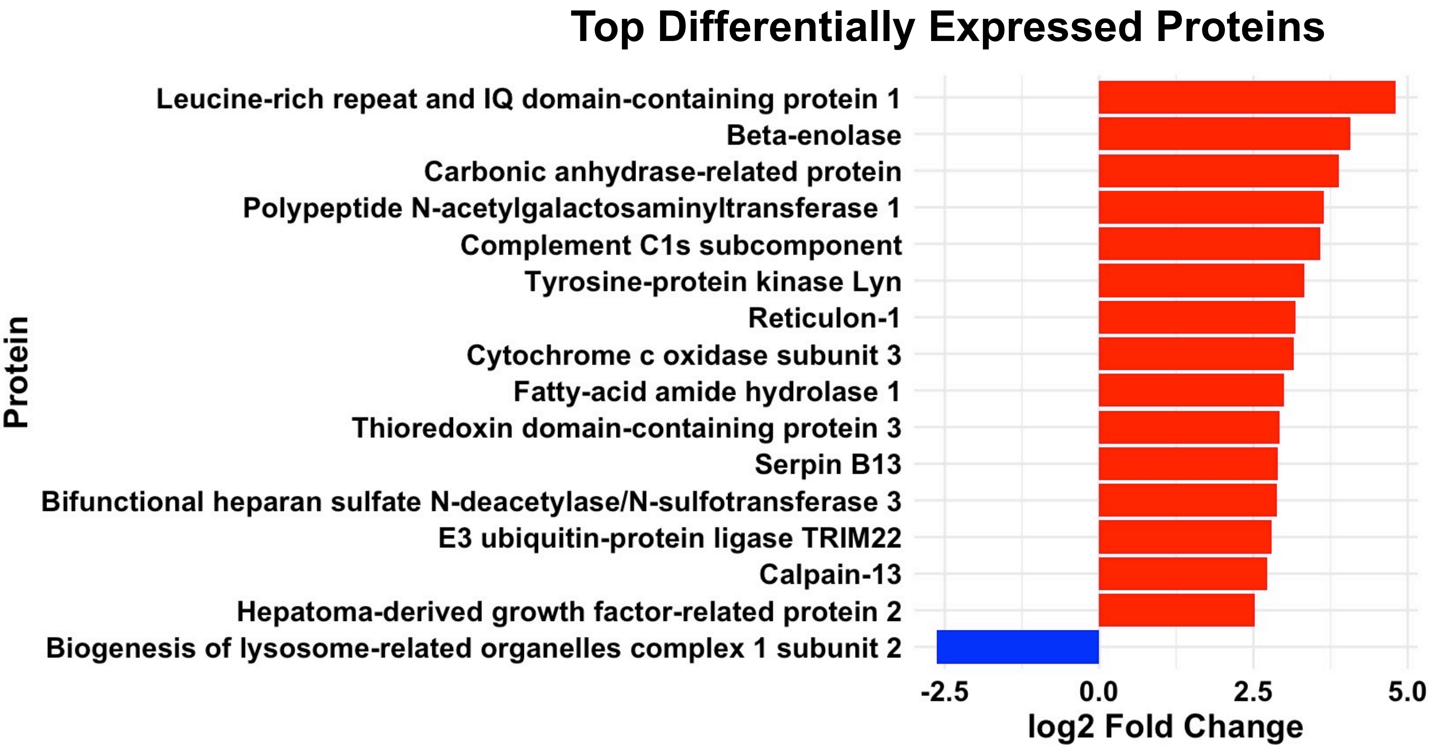

Top Canonical Pathways (z < 2)

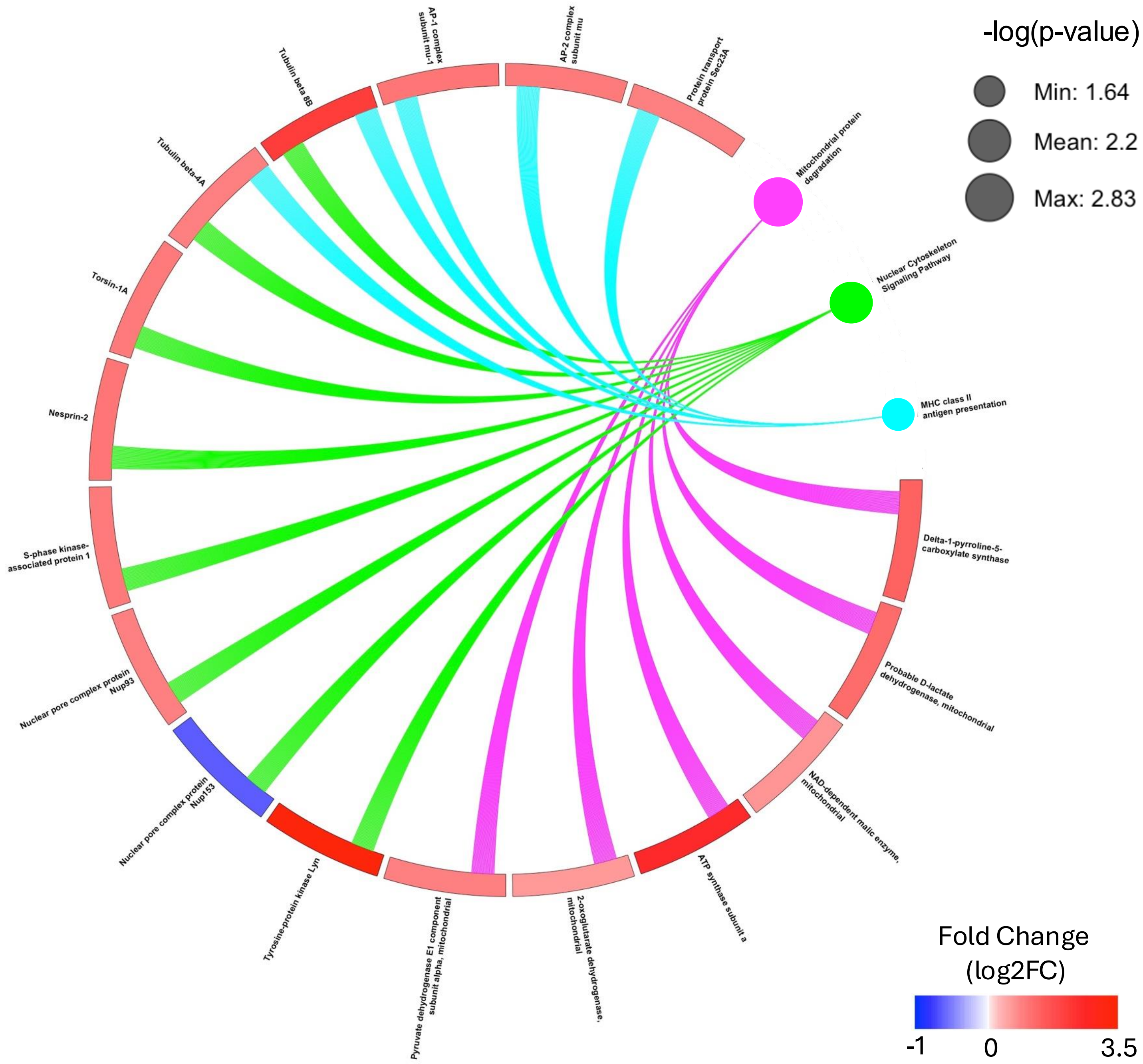

Supplement: Supplement 1 — Supplemental Figure 1: (A) Concentrations of hydroxyacetone quantified in VitroCell™ Cloud Chamber wells, these were the concentrations of hydroxyacetone that the individual inserts received in a single exposure. (B) Top differentially expressed proteins from hydroxyacetone treatments. Supplemental Figure 2: Circle plot connecting top IPA Canonical Pathways (z > 2) connected to DEPs in each pathway. Fold change of proteins is displayed by the protein name (red = upregulated, blue = downregulated) and –log(p-value) is reflected based on size of the dot by the pathway. [file media-1.pdf]
